# Time of year, age class and body condition predict Hendra virus infection in Australian black flying foxes (*Pteropus alecto*)

**DOI:** 10.1017/S0950268819001237

**Published:** 2019-07-10

**Authors:** D. Edson, A. J. Peel, L. Huth, D. G. Mayer, M. E. Vidgen, L. McMichael, A. Broos, D. Melville, J. Kristoffersen, C. de Jong, A. McLaughlin, H. E. Field

**Affiliations:** 1 Biosecurity Queensland, Department of Agriculture and Fisheries, Coopers Plains, Queensland, Australia; 2 Department of Agriculture, Canberra, ACT, Australia; 3 Environmental Futures Research Institute, Griffith University, Nathan, Queensland, Australia; 4 Medical Research Council, University of Glasgow Centre for Virus Research, Glasgow, UK; 5 EcoHealth Alliance, New York, NY, USA; 6 School of Veterinary Science, The University of Queensland, Gatton, Queensland, Australia

**Keywords:** Emerging infections, equine disease, infectious disease epidemiology, public health-emerging infections, zoonoses

## Abstract

Hendra virus (HeV) continues to cause fatal infection in horses and threaten infection in close-contact humans in eastern Australia. Species of *Pteropus* bats (flying-foxes) are the natural reservoir of the virus. We caught and sampled flying-foxes from a multispecies roost in southeast Queensland, Australia on eight occasions between June 2013 and June 2014. The effects of sample date, species, sex, age class, body condition score (BCS), pregnancy and lactation on HeV antibody prevalence, log-transformed median fluorescent intensity (lnMFI) values and HeV RNA status were assessed using unbalanced generalised linear models. A total of 1968 flying-foxes were sampled, comprising 1012 *Pteropus alecto*, 742 *P. poliocephalus* and 214 *P. scapulatus*. Sample date, species and age class were each statistically associated with HeV RNA status, antibody status and lnMFI values; BCS was statistically associated with HeV RNA status and antibody status. The findings support immunologically naïve sub-adult *P. alecto* playing an important role in maintaining HeV infection at a population level. The biological significance of the association between BCS and HeV RNA status, and BCS and HeV antibody status, is less clear and warrants further investigation. Contrary to previous studies, we found no direct association between HeV infection and pregnancy or lactation. The findings in *P. poliocephalus* suggest that HeV exposure in this species may not result in systemic infection and virus excretion, or alternatively, may reflect assay cross-reactivity with another (unidentified) henipavirus.

## Introduction

First described in 1994 [1], Hendra virus (HeV) (*Henipavirus: Paramyxoviridae*) continues to cause fatal equine infection and pose a threat of infection in close-contact humans in eastern Australia [2–4]. As of 30 April 2019, there have been 103 confirmed or possible equine cases and seven human cases of HeV reported, with case fatality rates around 90% and 60%, respectively [5]. Equine cases occur near-annually; the most recent human case was in 2009, putatively reflecting heightened and targeted awareness and risk mitigation communication by animal and public health authorities. In addition, two canine infections (both associated with equine cases) have been reported [6]. *Pteropus* spp. bats (colloquially known as flying-foxes in Australia) are the natural host of the virus [7–9], with *P. alecto* and *P. conspicillatus* shown to be the primary reservoir [10–12].

An effective vaccine for horses is available [13], and vaccination is regarded by animal health authorities as the single most effective means of preventing infection [14]. However, vaccine uptake has been limited, and minimizing contact between horses and flying-foxes remains a primary risk management strategy for many horse owners [15, 16]. To be effective, such strategies need to be underpinned by a comprehensive understanding of drivers and dynamics of HeV infection in flying-foxes [9, 17, 18].

Early studies of the ecology of infection in flying-foxes focused on individual animal serology and demonstrated that neutralizing antibodies to HeV were taxonomically and geographically widespread in flying-foxes [19]. Various studies have shown a higher antibody prevalence in older flying-foxes, in pregnant and lactating flying-foxes, and in black flying-foxes (*Pteropus alecto*) [19–22]. Later studies sought viral RNA using qRT PCR on pooled urine samples collected under roosting flying-foxes. These studies progressively demonstrated the limited genetic diversity of HeV [23], spatial and temporal heterogeneity in strain occurrence [23], higher probability of detection in black and spectacled flying-foxes [24], and spatiotemporal patterns of excretion [9, 25]. More recently, qRT PCR approaches have been used in individual animal studies, confirming black flying-foxes as an epidemiologically significant host species, and establishing urine as a primary route of excretion [11, 12].

While these studies have cumulatively provided information on which to base and refine exposure risk management strategies, the relative infection risk posed by various flying-fox cohorts over time, and the relationship between infection status and antibody status, has remained an enduring knowledge gap. In this longitudinal study, we describe, compare and contrast both serologic and molecular findings in samples from individual bats to better understand HeV infection and immune dynamics in flying-foxes and to better inform equine exposure risk management. Our aim is to identify key epidemiological variables significantly associated with HeV infection in flying-foxes.

## Methods

### Ethics statement

Fieldwork was approved under the (then) Queensland Department of Agriculture, Fisheries and Forestry Animal Ethics Committee Permit SA 2011/12/375 and the Queensland Department of Environment, Heritage and Protection Scientific Purposes Permits WISP05810609 and WISP14100614. Capture and sampling were undertaken by trained and experienced teams including at least one veterinarian.

### Capture and sampling

**Fig. 1.**
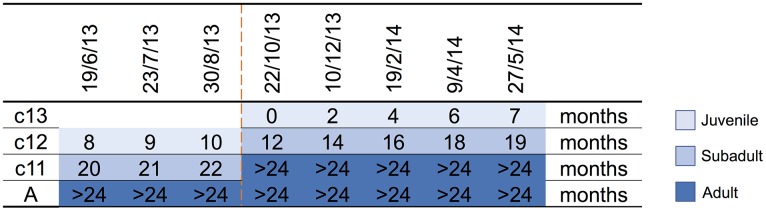
Birth cohort profiles over the study period, with sampling event midpoints and the age of sampled animal in months indicated. c13 = 2013 birth cohort, c12 = 2012 birth cohort, c11 = 2011 birth cohort, A = adult, and includes animals borne in the 2010 and earlier birth cohorts. The dashed line indicates the October mid-point of the annual birth pulse.

A parkland roost in the town of Boonah in southeast Queensland (27.992°S, 152.681°E) was purposively selected as the sampling location for several related studies because of its apparent permanency, substantial size, species diversity, accessibility and proximity to the Biological Sciences Laboratory (BSL) in Brisbane. Between June 2013 and June 2014, flying-foxes were non-randomly captured and sampled approximately every 2 months as described by Edson *et al*. [12]. Briefly, animals were captured using mist nets as they returned to roost pre-dawn; each animal was promptly extracted from the net and placed individually in a clean pillow case tied to a horizontal line pending anaesthesia and sample collection. Each bat was subsequently anaesthetised using the inhalation agent isoflurane and medical oxygen [26]. Capture and sampling events typically occurred over 7–10 successive days, with the mid-point taken as the sample date for analytical purposes. Species, sex, age class (juvenile, sub-adult, adult), bodyweight (g), forearm length (mm), body condition score (BCS) (1–5) and reproductive status (palpably pregnant, lactating) were recorded. Age class was based on morphometrics and sexual maturity, with juvenile <12 months, sub-adult 12–24 months and adult >24 months [12]. BCS was based on palpated pectoral muscle mass, with 1 = poor, 2 = less than fair, 3 = fair, 4 = greater than fair, 5 = good [12]. Bats were also classified by birth year cohort around the October mid-point of the annual birth pulse [27] (Fig. 1). Thus, we sampled bats born before 30 September 2011 (adults), bats born in the 2011 birth cohort (c11) which were ~20 months (sub-adults) at the beginning of the study and transitioned to adults during the study, bats born in the 2012 birth cohort (c12) which were ~8 months (juvenile) at the beginning of the study and ~19 months (sub-adults) at the end of the study, and bats born in the 2013 birth cohort (c13) which were born during the study period and ~7 months (juvenile) at the end. Adult bats could not be assigned a birth year cohort because of the limitations of ageing techniques based on morphometrics and sexual maturity beyond 24 months. One millilitre of blood was collected from the wing (cephalic) vein of each bat using a 25 G needle and 2 ml syringe, and immediately placed in a serum tube (1.3 ml BD Serum Tube^®^). Blood was allowed to clot for 6–24 h prior to centrifugation and separation of serum and packed haemocytes. Urine was collected into one or more plain 2 ml cryovials by gentle trans-abdominal compression of the bladder, and samples retained on ice prior to processing. Nasal, oral and rectal swabs (551C Copan^®^ Minitip Flocked Swab) (plus vaginal or preputial swabs when a urine sample was not obtained) were taken, immediately placed in 500 µl of phosphate-buffered saline (PBS) and kept on ice prior to processing. All bats were monitored post-anaesthesia until they regained consciousness, and after a further recovery period of 30–60 min, released at the point of capture.

*P. alecto* were preferentially targeted to facilitate investigation of infection dynamics because of the reported higher HeV detection rate in this species [9, 11, 12]. *P. poliocephalus* and *P. scapulatus* were included to enable comparative sero-epidemiology.

### Sample processing and testing

#### Serologic testing for anti-HeV antibodies

Serology was performed using a multiplex microsphere technique previously described by Bossart *et al*. [28]. Briefly, a pre-determined number of magnetic carboxylated microsphere beads (Fisher Biotec Pty Ltd, Australia) were selected for a protein coupling reaction. The HeV soluble G protein [29] was coupled to the bead set and mixed with the test sera at a dilution of 1:100. Bound antibody was detected using biotinylated Protein A at 1:500 and biotinylated Protein G at 1:250 (Pierce, USA); the reporter streptavidin-phycoerythrin was added at 1:1000 (Qiagen Pty Ltd, Australia). The beads were analysed using a Luminex MAGPIX system, and results were recorded as median fluorescent intensity (MFI).

The likelihood that an individual MFI value was positive or negative was determined by calculating cut-offs using Bayesian mixture models following Peel *et al*. [30] and Chowdhury *et al*. [31]. However, after log-transformation of MFI values, the seronegative and seropositive distributions remained positively and negatively skewed, respectively, indicating that normal distribution models were inappropriate. The mixture model was therefore fitted with a shifted-Gompertz and a Gompertz distribution in R [32]. Conservative species-specific cut-offs were calculated (*P. alecto*, 1636; *P. poliocephalus*, 992; *P. scapulatus*, 1339) so that individuals with MFI values above the cut-off had a probability in excess of 95% of having anti-HeV antibodies. Natural logarithm MFI (lnMFI) values have previously been found to correlate strongly with neutralizing antibody titre [28, 30, 33], and are a robust indication of immune dynamics at a population level. Further details, including potential limitations of this assay, are provided as Supplementary information (Text S1, Table S1, Figs S1 and S2).

#### Molecular testing for HeV RNA

The quantitative RT-PCR data reported in Edson *et al*. [12] are re-analysed in this paper after pairing with serological data. Briefly, they added 50 µl of urine, serum, packed haemocytes and PBS from nasal, oral and rectal swab samples (plus vaginal or preputial swabs when a urine sample was not obtained) individually to 130 µl of lysis solution (MagMAX, Ambion, Texas, Cat AM8500) to inactivate virus particles and preserve RNA for PCR screening. Total nucleic acid was extracted using a magnetic bead-based nucleic acid extraction kit (MagMAX-96 viral RNA isolation system, Ambion, Texas, Cat AM1836-5) run on a magnetic particle handling system (Kingfisher KF-96, Thermo-Scientific, Finland) according to the manufacturer's instructions. Using the AgPath-ID One-Step RT-PCR Kit (Life Technologies, Melbourne, Australia), 5 µl of nucleic acid extract was added to 20 µl of mastermix. Forward and reverse primers and probe targeting a 69 base pair region on the M gene were used [34]. Positive and negative controls were included in each run. Assays were run on a 7500 Fast Real-Time PCR System (Applied Biosystems) in standard mode for a total of 45 cycles in accordance with the manufacturer's instructions for the mastermix. As described in Edson *et al*. [12], a subset of individuals (621/1968) had all sample types tested; for the remainder, serum and urine (or vaginal or preputial swabs when urine was not obtained) were used as a screening panel. An individual was considered ‘RNA-positive’ if any sample type yielded a positive PCR result (a Ct value <40), and ‘RNA-negative’ otherwise [34]. However, Edson *et al*. [12] found that only 9/16 individuals positive on their urine sample were positive on their paired vaginal or preputial swab, suggesting the RNA-negative cohort potentially contains a small number of false-negative results where individuals tested negative on vaginal or preputial swab in the absence of a urine sample (see Discussion). Thus, analysis of the PCR data was undertaken at three levels: animal (individuals with at least one sample type tested), urine (only individuals with a urine sample) and serum (only individuals with a serum sample).

#### Statistical methods

The effects of sampling date, species, sex, age, BCS, pregnancy and lactation, and their respective interactions, on HeV antibody prevalence, MFI and HeV RNA status were assessed, with the exception that species were excluded from the latter as all RNA detections were in a single species (*P. alecto*). Data for each variable were subjected to an unbalanced generalised linear model [35] using GenStat 2013 [36]. The binomial distribution and logit link were adopted for binary data (HeV antibody prevalence and RNA status) and the ln-Normal for MFI data. The initial model included the design variables sampling event midpoint date (sample date), age class, species, sex and the interaction term date.age, with additional models separately adding BCS (as a linear contrast), pregnancy and lactation (as these were correlated). All models were repeated using age cohort instead of age class. The exact binomial method for confidence intervals [37] was adopted for zero prevalence values, as the logit estimates here become unstable. All significance testing was conducted at the 95% level (*P* < 0.05). Adjusted means (marginal, i.e. weighted by the numbers of observations in each class) and standard errors were estimated for each variable, along with 95% confidence intervals (calculated on the logit scale and back-transformed to percentages). The residual plots for most variables proved to be approximately normal. Those that showed positive skewness and heterogeneous variance were transformed using the natural logarithm (ln).

*P. scapulatus* were excluded from the serologic analysis as the absence of a clear bimodal distribution of lnMFI values and a suboptimal model fit precluded robust interpretation for this species (Text S1, Table S1, Figs S1 and S2).

## Results

A total of 1968 flying-foxes were captured and sampled over the 13-month study period, comprising 1012 *P. alecto*, 742 *P. poliocephalus* and 214 *P. scapulatus* (Fig. S3). PCR results were obtained for all individuals; serology results were obtained for 1906 individuals (967 *P. alecto*, 734 *P. poliocephalus*, 205 *P. scapulatus*). Model outputs and sample sizes of variables significantly associated with HeV RNA detection and antibody detection are presented in Data S1, Table S2 and Table S3, respectively.

### RNA prevalence

**Fig. 2.**
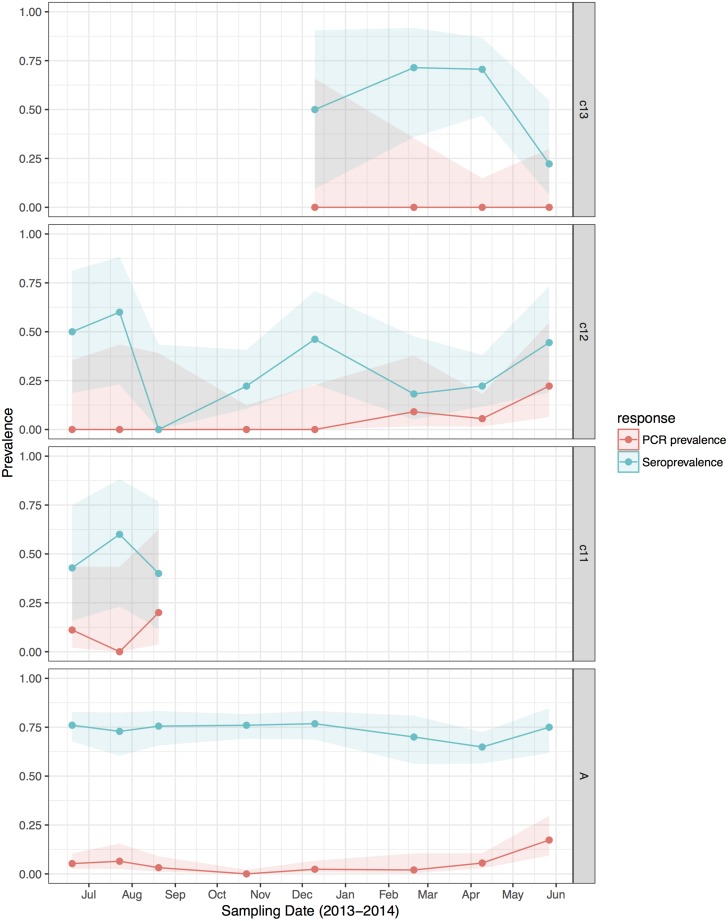
Hendra virus RNA and antibody prevalence in *P. alecto*, with juvenile and sub-adult data presented as birth-year cohorts. c13 bats were born in the 2013 birth season and were ~7 months old at the end of the study; c12 bats were born in the 2012 birth season and were ~8 months old at the start of the study and ~19 months old at the end of the study; c11 bats were born in the 2011 birth season and were ~20 months old at the start of the study, and joined the adult (a) cohort during the study period.

**Fig. 3.**
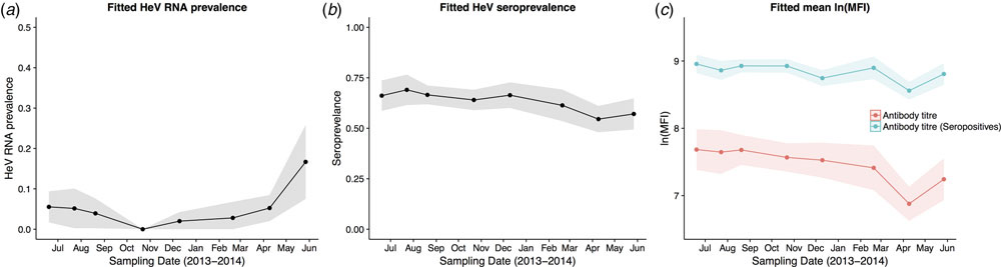
Model fitted Hendra virus RNA prevalence in *P. alecto* (a), mean anti-Hendra virus antibody prevalence in *P. alecto* and *P. poliocephalus* (b), and mean anti-Hendra virus lnMFI in *P. alecto* and *P. poliocephalus* (c).

HeV RNA was detected only in *P. alecto*, with 42 of 1012 individuals (4.2%, 95% CI 3.1–5.6%) positive on one or more sample type, including serum (11/1000), urine (26/558), vaginal and preputial swabs (18/871), rectal swabs (8/318), nasal swabs (3/306) and oral swabs (2/307) [12]. None of the 2013 birth cohort (~0–7 months during the study period, *n* = 35) tested positive. The youngest bats that tested positive for RNA were sub-adults ~16 months of age (2012 birth cohort), with cohort prevalence increasing to the end of the study when the bats were ~19 months. Limited data points precluded the meaningful interpretation of the 2011 cohort (Fig. 2), but a similar temporal pattern to that seen in sub-adults was evident in the adult cohort. RNA prevalence varied significantly with sample date (peaking mid-year, Fig. 3a) and age class (sub-adults > adults) in the initial model, and with BCS (poor > fair > good), and antibody status (positive > negative) in additional models (Table 1). Of 38 bats that were RNA-positive and had matched antibody data, 34 were antibody-positive and four were antibody-negative. There was no association between RNA prevalence and sex, pregnancy or lactation, although prevalence differed slightly among adult *P. alecto* that were pregnant (14/195, 7.2%, 95% CI 4.3–11.7), lactating but not pregnant (3/132, 2.3%, 95% CI 0.8–6.5) or were neither pregnant nor lactating (5/96, 5.2%, 95% CI 2.2–11.6). RNA detection in urine was positively associated with sample date and antibody status (positive > negative), but not BCS; RNA detection in serum was positively associated with sample date, age class (sub-adult>adult) and sex (female>male), but not BCS or antibody status. None of the 742 *P. poliocephalus* or the 214 *P. scapulatus* yielded a positive PCR result on any sample [12], yielding theoretical upper 95% confidence intervals for infection prevalence for these species of 0.5% and 1.7%, respectively.

**Table 1.** Variables significantly associated with molecular and serological measures of HeV infection^a^ in wild-caught flying-foxes sampled at Boonah in southeast Queensland in 2012–2013

	HeV RNA			HeV antibody		
	Detection (animal)	Detection (urine)	Detection (serum)	Detection (serum)	lnMFI value (serum)	lnMFI value (antibody-positive animals)
Sample size	1012	558	1000	1701	1701	1072
Sample date^b^	***	**	*	***	***	***
Age class^b^	*	NS	*	***	***	**
Species^b^				***	***	***
Sex^b^	NS	NS	*	NS	NS	***
Date.age^b^				**	*	
BCS	**	NS	NS	**	***	NS
Pregnancy	NS	NS	NS	NS	NS	NS
Lactation	NS	NS	NS	NS	NS	NS
Age cohort	NS	NS	NS	***	***	***
HeV antibody status	***	**	NS			
HeV RNA status					***	***

NS, not significant, *<0.05, **<0.01, ***<0.001, blank, association not tested.

a HeV RNA models included data from *P. alecto* only, as *P. poliocephalus* did not yield any PCR-positive samples. HeV antibody models included data from both *P. alecto* and *P. poliocephalus* samples.

b Initial model variables.

Full results of all models are provided as Supplementary information.

### Antibody prevalence

**Fig. 4.**
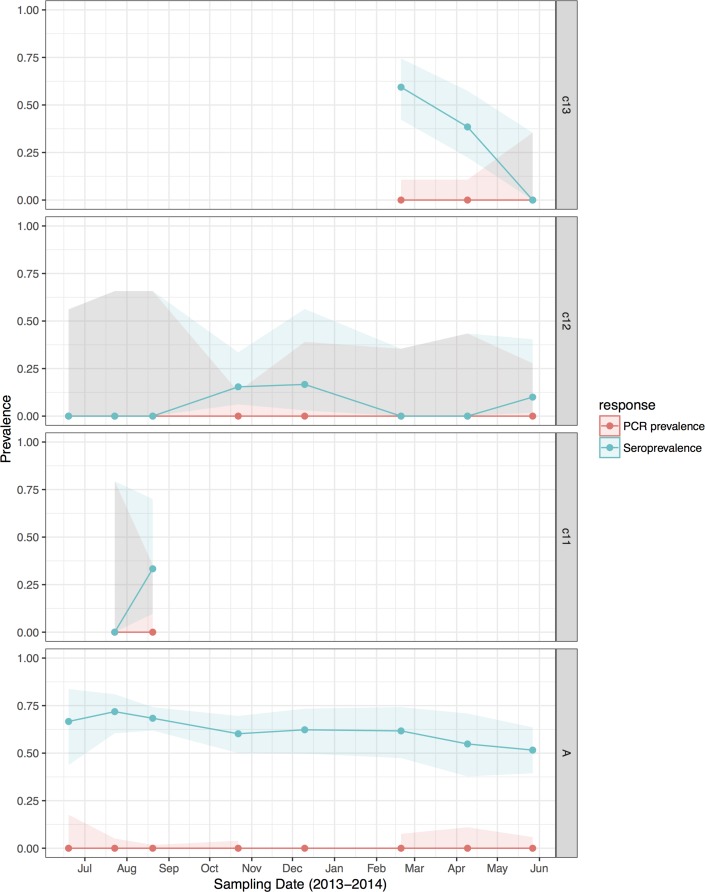
Hendra virus RNA and antibody prevalence in *P. poliocephalus*, with juvenile and sub-adult data presented as birth-year cohorts. c13 bats were born in the 2013 birth season and were ~7 months old at the end of the study; c12 bats were born in the 2012 birth season and were ~8 months old at the start of the study and ~19 months old at the end of the study; c11 bats were born in the 2011 birth season and were ~20 months old at the start of the study, and joined the adult (A) cohort during the study period.

HeV antibody was detected in both *P. alecto* (651/967) and *P. poliocephalus* (421/734). Antibody prevalence varied significantly with sample date (declining over the study period) (Fig. 3b), age class (adult > juvenile > sub-adult), species (*P. alecto* > *P. poliocephalus*) and the interaction term date.age in the initial model, and with cohort (adult > c13 > c11 > c12) and BCS (good > fair > poor) in additional models (Table 1). For both *P. alecto* and *P. poliocephalus*, antibody prevalence was relatively static in adults, but more dynamic in juveniles and sub-adults as illustrated in Figure 2 (*P. alecto*) and Figure 4 (*P. poliocephalus*). In the 2013 cohort, prevalence in both species declined by the end of the study when these individuals were approximately 7 months of age. In the 2012 cohort, antibody prevalence in both species was 0% at ~10 months of age (*P. alecto* 0/80, 0%, 95% CI 0.0–4.6; *P. poliocephalus* 0/35, 0%, 95% CI 0.00–9.9), then increased across the next two sample dates before fluctuating. Limited data points for the 2011 cohort again precluded meaningful interpretation. There was no association between antibody prevalence and sex, pregnancy or lactation. In adult *P. alecto*, prevalence did not differ significantly among individuals that were pregnant (143/184, 77.7%, 95% CI 71.2–83.1), lactating but not pregnant (105/129, 81.5%, 95% CI 73.8–87.2) *vs.* those that were neither pregnant nor lactating (66/92, 71.7%, 95% CI 61.8–79.9). Similarly, antibody prevalence in adult *P. poliocephalus* did not differ significantly with pregnancy or lactation status, but was lower than in adult *P. alecto* across all reproductive stages; pregnant (105/165, 63.6%, 95% CI 56.0–70.6.1), lactating but not pregnant (33/57, 57.9%, 95% CI 45.0–69.8), neither pregnant nor lactating (31/62, 50.0%, 95% CI 37.9–62.1).

Comparative summary viral RNA and antibody prevalence data for *P. alecto* are presented in Table 2.

**Table 2.** Comparative Hendra virus molecular and serology findings in 967^a^ *P. alecto* sampled at Boonah between June 2013 and June 2014

	Animals	Numbers (%) of animals			
		RNA +ve, antibody +ve	RNA +ve, antibody −ve	RNA −ve, antibody +ve	RNA −ve, antibody −ve
Adult	803	29 (3.6)	2 (0.2)	562 (70)	210 (26.2)
c11	17	2 (11.7)	0 (0)	6 (35.3)	9 (53)
c12	112	3 (2.7)	2 (1.8)	29 (25.9)	78 (69.6)
c13	35	0 (0)	0 (0)	20 (57.1)	15 (42.9)
Totals	967	34 (3.5)	4 (0.4)	617 (63.8)	312 (32.3)

a Nine hundred sixty-seven *P. alecto* had positive results for both PCR and serology. While 734 *P. poliocephalus* had positive serology, none had positive PCR results, precluding their inclusion here.

### lnMFI value

A significant association was observed between lnMFI and sample date (Table 1, Fig. 3c), age class (adult > juvenile > sub-adult), species (*P. alecto* > *P. poliocephalus*) and the interaction term date.age, and with cohort (adult > c13 > c11 > c12), BCS (good > fair > poor) and RNA status (positive > negative) in additional models. In *P. alecto*, lnMFI values in adults and sub-adults were highest in winter, and in juveniles, highest in summer. There were no significant associations with sex, pregnancy or lactation in adult *P. alecto* (Fig. S4) or *P. poliocephalus* (Fig. S5).

**Fig. 5.**
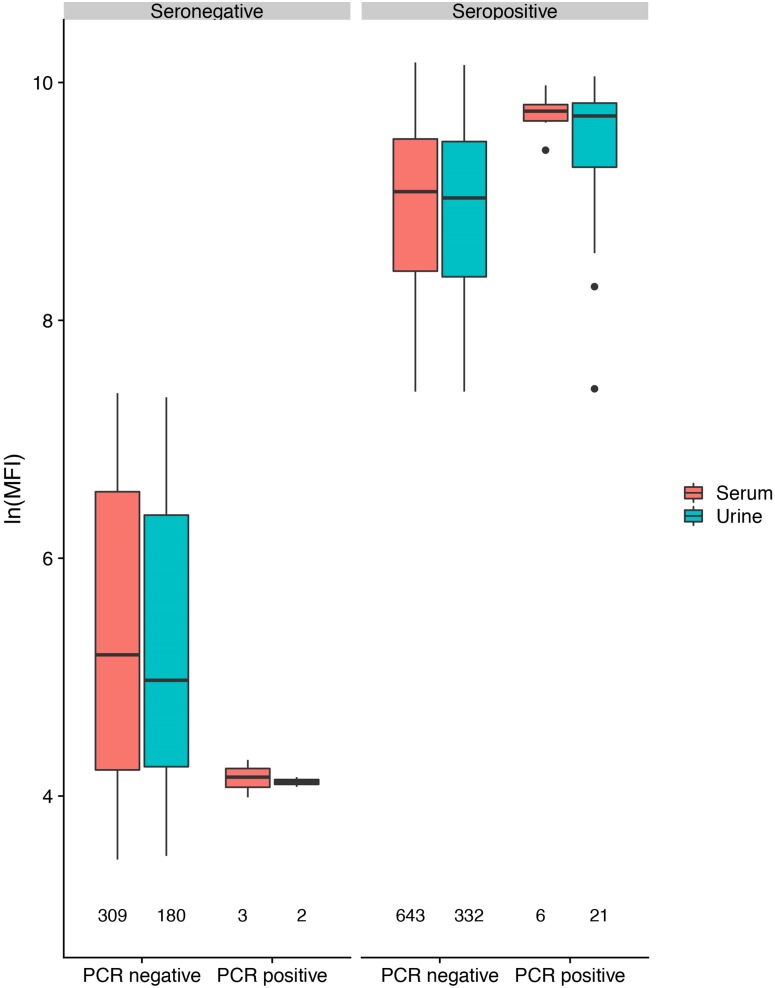
Hendra virus antibody lnMFI values in *P. alecto* individuals by HeV RNA detection status in serum or urine.

In antibody-positive animals, lnMFI values were significantly associated with sample date, age class (adult>sub-adult>juvenile), species (*P. alecto* > *P. poliocephalus*) and sex (female > male) in the initial model, and with cohort (c12 > adult > c13 > c11) and RNA status (positive > negative) in additional models (Table 1, Fig. 3c, Fig. S6). Of 11 *P. alecto* individuals with HeV RNA detected in serum (seven adult females, three sub-adult females and one sub-adult male), nine also had concurrent antibody data, and seven also had concurrent urine RNA data. Six of the former were antibody-positive, with high lnMFI values (mean lnMFI = 9.7) compared to RNA-negative individuals (mean lnMFI = 7.8) and to the majority of urine RNA-positive individuals (mean lnMFI = 9.4) (Fig. 5). The remaining three serum RNA-positive bats (33%) were antibody-negative, compared to only 8.7% (2/23) of urine RNA-positive bats testing antibody-negative. Of six individuals with urine and serum RNA data plus antibody data, four were concurrently positive across all three tests (all adult females), one was serum RNA-positive and antibody-positive, but urine RNA-negative (adult female), and one was urine and serum RNA-positive, but antibody-negative (sub-adult male).

## Discussion

This study sought to identify epidemiological variables significantly associated with HeV infection in a mixed-species colony of free-living flying-foxes over time. With a sample size of 1968 individuals, we sought associations with both RNA status as a measure of current infection, and antibody status and lnMFI values as measures of past infection. To our knowledge, this is the first study to examine concurrent serologic and molecular data of individual flying-foxes, and thus gain an insight into both infection and immune dynamics.

The molecular data used in this analysis has been previously analysed in the context of routes of HeV excretion [12] and showed that HeV RNA detection was limited to *P. alecto*, with no detection in *P. poliocephalus* or *P. scapulatus*. Here, we focus on identified epidemiological associations with HeV RNA detection in *P. alecto*. We found that RNA detection varied over the year, with infection in the colony more likely in the autumn and winter months. This finding is consistent with those of Field *et al*. [9] across the broader southeast Queensland/northeast NSW region, and with the winter clustering of equine cases in southeast Queensland/northeast NSW [38, 39], although a temporal association with infection (in flying-foxes and horses) appears to be weaker or lacking or beyond this region. The positive association between RNA detection and sub-adults in the 2012 birth cohort is consistent with a seasonal pulse of susceptible individuals as maternal HeV antibodies wane [40] and suggest sub-adults may play an important role in maintaining HeV infection at a population level. This scenario has previously been hypothesised in relation to HeV [41], and parallels exist in relation to other infectious diseases [42–44]. However, given the complex nature of the system, it is likely that additional drivers (e.g. population dynamics, environmental factors) also influence HeV transmission in flying-foxes [9, 25].

The observed positive association between RNA detection and antibody detection, as well as the higher lnMFI values in RNA-positive individuals, is consistent with seroconversion during infection (or at least before viral clearing), and with seroconversion (and boosted lnMFI values) associated with recurring or persistent infection. Individuals testing positive for HeV RNA in serum were less likely to be concurrently seropositive than individuals testing positive for RNA in urine, but when they were seropositive, they had higher lnMFI values on average. This could suggest that in natural infections, RNA in the peripheral circulation is indicative of very recent infection, with either insufficient time for a detectable IgG response to be mounted (seronegative), or rapid seroconversion to high lnMFI values (seropositive). These findings and interpretations contrast with earlier experimental studies which suggested that infection in serum and urine occurred simultaneously, and that antibody response was unpredictable and not reflective of time since infection [8]. Notwithstanding, the identified positive association between RNA detection and lnMFI values identified here suggests that recent/current infection provokes a strong IgG response in both sub-adult and adult bats. While the inability of the assay to detect IgM constrains further interpretation, it is plausible that RNA detection in sub-adults more likely represents their first infection, and their lnMFI values a primary response; with increasing age, adults are more likely to have been previously infected. More broadly, it is unclear whether the 26% adult bats that were both RNA-negative and antibody-negative have never been exposed to HeV, or whether they have been exposed but did not develop (or no longer have) detectable antibodies. Regarding the latter, it also remains unknown whether these bats are susceptible to re-infection, or whether primary infection provides lifelong protective immunity against re-infection in the absence of detectable antibodies, as has been suggested in other bat-viral systems [45].

The statistical association between positive RNA status and lower BCS in the animal-level analysis (but not the urine- or serum-level analyses) is of unknown biological significance. It may simply reflect correlation, with the probability of infection temporally coincident with seasonal weight loss [46]; alternatively, it could reflect causation, with individuals in poorer body condition more likely to become infected, or at least, more likely to excrete virus. The latter could plausibly include a scenario of recrudescing infection and viral shedding as body condition declines [21, 47]. However, interpretation should consider the possibility of a small number of individuals being misclassified as ‘RNA-negative’ on the basis of a negative vaginal or preputial swab where a urine sample was not available, as Edson *et al*. [12] found that negative vaginal or preputial swabs under-estimated RNA presence in urine by around 44%. Consistent with our study, McMichael *et al*. [48] found a significant association between positive HeV RNA status and lower triglyceride levels, a biomarker which they had previously shown to correlate with lower BCS [49], but no association in urine-only analyses between HeV status and the majority of other biomarkers strongly correlated with BCS.

The reverse association was found between antibody status and BCS, with animals in poorer condition less likely to have anti-HeV antibodies (and more likely to have lower lnMFI values) than animals in good condition. Immune responses are energetically costly; energy restriction, with or without malnutrition, can result in suppression of the immune system and increased infection and mortality rates [50]. While speculative, our findings may suggest that animals in poorer body condition are less capable of mounting an effective innate and/or humoral immune response, resulting in an increasing likelihood of infection and/or viral excretion. Kessler *et al*. [51] suggest that the combined effects of habitat loss and resource provisioning are driving changes in fruit bat feeding ecology and nutrition, and may be associated with increasing risk of viral spillover. Interestingly, the association between poorer body condition and a weaker serologic response (in terms of both serostatus and lnMFI values) was also observed in *P. poliocephalus* (data not shown) suggesting the same mechanism may be at play, but as previously stated, HeV RNA was not detected in this species.

The absence of any association between pregnancy or lactation and RNA detection, antibody status or lnMFI values, respectively, differs from previous serology-only studies. Plowright *et al*. [21] detected significantly higher antibody prevalence in late pregnant/early lactation female little red flying foxes (*P. scapulatus*) compared with males or non-reproductive females. Breed *et al*. [22] detected higher antibody prevalence in pregnant and lactating spectacled flying foxes (*P. conspicillatus*), with titres significantly elevated in pregnant animals. Nonetheless, we did find two notable (and possibly linked) significant associations that suggest the role of female bats in the ecology of HeV infection warrants further focus; namely seropositive females had higher lnMFI values than seropositive males, and viral RNA was more likely to be detected in serum in females than males (Table 1). The latter finding has also been reported in experimentally infected *P. alecto* [8]. While our study had large sample sizes overall, high pregnancy rates in adult female bats mean that sample sizes for non-pregnant/non-lactating controls at each time point are limited, constraining the robust interpretation of the effect of sex, pregnancy and lactation from broader seasonal drivers. Specific studies to investigate the mechanisms for such interactions are warranted.

*P. alecto* had a higher mean antibody prevalence and lnMFI values than did *P. poliocephalus*. While not unexpected given increasing evidence that the former is a primary reservoir host, the detection of anti-HeV antibodies in over 50% of *P. poliocephalus* is intriguing, in that it suggests that these individuals have been sufficiently exposed to HeV to mount a humoral response. Yet, in a colony in which HeV is circulating, evidenced by RNA detection in multiple *P. alecto*, and with nearly half the *P. poliocephalus* cohort evidently susceptible as indicated by their antibody-negative status, viral RNA was not detected in any *P. poliocephalus* over the entire study period. Given the epidemiologically ‘adequate’ contact between individuals of the two species when co-roosting, and daily contact with potentially infectious urine [12], plus the substantial sample size, it is highly improbable that the zero detection in *P. poliocephalus* is a chance event – the upper 95% confidence interval for the population infection rate is 0.5%. The same rationale could be applied to the lack of RNA detection in *P. scapulatus*, though the argument in this species is somewhat constrained by the smaller sample size, and the uncertain validity of the serology in this species. Targeted studies are required to address variable host species’ responses to henipavirus infection and the various hypotheses put forth below. A plausible explanation, at least in *P. poliocephalus*, is that the nature of exposure or infection may be adequate to provoke an innate immune response and antibody production, so that the virus is cleared without systemic viral replication and excretion; for example, localised infection and replication of mucous membranes. This scenario is consistent with arguments proposed by Dups *et al*. [52] and Goldspink *et al*. [11] to explain the aspects of HeV infection dynamics. An alternative interpretation is that the antibody response detected in *P. poliocephalus* reflects assay cross-reactivity with a closely related henipavirus [53]. This interpretation could explain the lower lnMFI values (reflecting lower binding specificity) observed in *P. poliocephalus* and the lack of HeV RNA detection even in low BCS *P. poliocephalus* as discussed above. However, lower lnMFI values in *P. poliocephalus* might also plausibly reflect a less efficient humoral response associated with non-systemic infection.

The reason for a lack of a clear bimodal distribution of *P. scapulatus* lnMFI values is unknown, however could suggest cross-reactivity within the serological assay with an unknown Hendra-like virus, a diverse exposure history to HeV within the population, or less plausibly reflect the smaller sample size for this species in this study. Cross-reactivity is a plausible explanation for the detection of anti-HeV antibodies in *P. scapulatus* given the recent detection of novel henipaviruses in this species [53] and the repeated lack of detection of HeV RNA using a qRT-PCR specific for HeV. Future studies aimed at isolating henipaviruses from *P. scapulatus* populations are required to clarify this.

Our study is novel in its concurrent molecular and serologic screening of a sample of 1906 bats for evidence of HeV infection, and the longitudinal design has allowed us to explore and explain within-year associations at both event and individual levels with confidence. However, the lack of replicates over multiple years means that inter-annual generalisations should be made with care.

## Conclusion

The study has yielded significant new insights into HeV infection and immune dynamics in flying-foxes, as well as provided additional support for a number of previous hypotheses. In summary, our findings support immunologically naïve sub-adult *P. alecto* playing an important role in maintaining HeV infection at a population level. Contrary to previous studies, we found no association between HeV infection and pregnancy or lactation, and therefore no support for reproductive stress as a driver for infection or recrudescing infection associated with pregnancy. The paradoxical serologic and molecular findings in *P. poliocephalus* suggest that HeV exposure in this species may not routinely result in systemic infection and virus excretion, or alternatively, that the serologic findings reflect assay cross-reactivity with another henipavirus. Finally, we identify an association between BCS and infection that plausibly could support a role for immune system competence in HeV infection in flying-foxes.

## Supplementary material

For supplementary material accompanying this paper visit https://doi.org/10.1017/S0950268819001237. click here to view supplementary material
