# Supplementary material for: Time of year, age class and body condition predict Hendra virus infection in Australian black flying foxes (Pteropus alecto)
Source: Epidemiol Infect. 2019 Jul 10;147:e240. doi: 10.1017/S0950268819001237 (PMC6625375; doi:10.1017/S0950268819001237)
Supplement: Supplementary file 1 [file S0950268819001237sup001.docx]

**Epidemiology and Infection**

Time of year, age class and body condition predict Hendra virus infection in Australian black flying foxes (*Pteropus alecto*).

D. Edson, A. J. Peel, L. Huth, D. G. Mayer, M. E. Vidgen, L. McMichael, A. Broos, D. Melville, J. Kristoffersen, C. de Jong, A. McLaughlin and H. E. Field.

**Supplementary material**

**Supplementary Text S1:** Bayesian Mixture model

Methods:

Peel et al, 2013 (1) used a Bayesian mixture model was used to characterize the bimodal distribution of microsphere assay outputs to classify individuals as seropositive or seronegative. The authors assumed that detection of antibodies when they are present is uniform across individuals within a species, resulting in the same underlying ‘seropositive’ or ‘seronegative’ distributions across age classes. Differences in the observed distributions among age classes or populations are therefore explained solely by changes in proportions seropositive/seronegative.

This was extended to a multi-species study in Chowdhury et al, 2014 (2), however, optimal fitting was observed when each species was fitted independently. This approach was taken in our study, where we fitted serological data from black flying foxes (*Pteropus alecto*), grey-headed flying foxes (*P. poliocephalus*) and little red flying foxes (*P. scapulatus*) separately.

Initial data exploration indicated that even after log-transformation of titres (mean fluorescence intensity, MFI), the seronegative and seropositive distributions were positively and negatively skewed, respectively, and normal distribution models were inappropriate. The mixture models were therefore fitted with a shifted-Gompertz and a Gompertz distribution in R (3) using packages Rcpp (4) and coda (5).

Results:

Model convergence, autocorrelation and the Monte Carlo error for the posterior mean were assessed as per Peel et al, 2013 (1), and determined as satisfactory. The fitted distributions (Figure S1) indicated good model fit for black flying fox (BFF) and grey-headed flying fox (GHFF) data, however the underlying distribution was not clear for little red flying fox (LRFF) data and the overall fit was suboptimal.

Species-specific cutoffs were estimated so that individuals with MFI values above this cutoff were >99% likely to be seropositive (Table S1 and Figure S2). These values were derived as in Peel et al, 2013 (1). Cutoff estimates were considered unreliable for LRFF data.

Limitations:

Virus neutralisation assays are the gold standard for measuring host response to infection. The primary limitations of bead-based binding assays are, firstly, that they use Protein A and Protein G to bind to antibodies, which only bind to IgG, not IgM; and secondly, that antibodies are only detected to these viral proteins, not whole virus. Nonetheless, natural logarithm MFI (lnMFI) values have previously been found to correlate well with neutralizing antibody titre (1, 6, 7), and at a population level, they provide a valid and robust indication of immune dynamics.

References:

(1) **Peel AJ, et al.** Use of cross-reactive serological assays for detecting novel pathogens in wildlife: assessing an appropriate cutoff for henipavirus assays in African bats. *Journal of Virological Methods* 2013; **193;** 295–303.

(2) **Chowdhury S, et al.** Serological Evidence of Henipavirus Exposure in Cattle, Goats and Pigs in Bangladesh. *PLoS Neglected Tropical Diseases* 2014; **8**: e3302.

(3) **R Core Team.** R: A language and environment for statistical computing. Version 3.0. 2016.

(4) **Eddelbuettel D, Francois R.** Rcpp: Seamless R and C plus plus Integration. *Journal of Statistical Software* 2011; **40:** 1–18.

(5) **Plummer M, et al.** CODA: Convergence Diagnosis and Output Analysis for MCMC. *R news* 2006; **6**: 7–11.

(6) **Bossart KN, et al.** Neutralization assays for differential henipavirus serology using Bio-Plex Protein Array Systems. *Journal of Virological Methods* 2007; **142**(1-2): 29-40.

(7) **Peel A, et al.** Henipavirus neutralising antibodies in an isolated island population of African fruit bats. *Plos One* 2012; **7**(1).

**Supplementary Table S1:** Posterior means and 95% credible intervals for the cutoff for each species so that individuals with MFIs above that cutoff are ≥95% likely to be seropositive.

|  | **Mean *ln*(MFI)** | **95% C.I.** | **Mean MFI** | **95% C.I.** |
| --- | --- | --- | --- | --- |
| BFF | 7.4 | (7.0 – 7.7) | 1636 | (1097 – 2208) |
| GHFF | 6.9 | (6.6 – 7.2) | 992 | (735 – 1339) |
| LRFF | 7.2 | (6.1 – *) | 1339 | (446 – *) |

* The upper credible interval of the likelihood of being seronegative for LRFF never reaches 5%, so the UCI of the cutotff is not estimable at this threshold.

**Supplementary Table S2:** Sample sizes of variables significantly associated with HeV RNA detection in wild-caught flying-foxes sampled at Boonah in southeast Queensland in 2012-13. RNA models included data from *P. alecto* only, as *P. poliocephalus* did not yield any PCR-positive samples. Accession numbers are in the format of ‘yymmdd’.

| **Variable** | **Category** | **RNA- positive** | **Total** |
| --- | --- | --- | --- |
| Accession | 130619 | 8 | 149 |
|  | 130723 | 4 | 72 |
|  | 130820 | 4 | 105 |
|  | 131022 | 0 | 204 |
|  | 131210 | 3 | 142 |
|  | 140219 | 2 | 68 |
|  | 140409 | 10 | 202 |
|  | 140527 | 11 | 70 |
| Age Cohort | J.13 | 0 | 40 |
|  | J.12 | 0 | 18 |
|  | SA | 7 | 115 |
|  | A | 35 | 839 |
| Sex | F | 27 | 529 |
|  | M | 15 | 483 |
| Body Condition score | 1 | 3 | 62 |
|  | 2 | 5 | 145 |
|  | 3 | 18 | 377 |
|  | 4 | 11 | 236 |
|  | 5 | 5 | 180 |
| Pregnant (Adult females) | Not Pregnant | 8 | 228 |
|  | Pregnant | 14 | 195 |
| Lactating (Adult females) | Not lactating | 18 | 282 |
|  | Lactating | 4 | 141 |
| Ab status | Seropositive | 4 | 316 |
|  | Seronegative | 34 | 651 |

**Supplementary Table S3:** Sample sizes of variables significantly associated with HeV antibody (Ab) detection in wild-caught flying-foxes sampled at Boonah in southeast Queensland in 2012-13. Antibody models included data from both *P. alecto* (BFF) and *P. poliocephalus* (GHFF) samples*.* Accession numbers are in the format of ‘yymmdd’.

|  |  | **BFF** | | **GHFF** | |
| --- | --- | --- | --- | --- | --- |
| **Variable** | **Category** | **Ab Positive** | **Total** | **Ab Positive** | **Total** |
| Accession | 130619 | 98 | 134 | 12 | 21 |
|  | 130723 | 49 | 69 | 51 | 74 |
|  | 130820 | 70 | 100 | 151 | 226 |
|  | 131022 | 139 | 202 | 60 | 119 |
|  | 131210 | 103 | 140 | 39 | 67 |
|  | 140219 | 42 | 68 | 48 | 86 |
|  | 140409 | 105 | 184 | 27 | 62 |
|  | 140527 | 45 | 70 | 33 | 79 |
| Age cohort | J.13 | 20 | 35 | 29 | 65 |
|  | J.12 | 6 | 16 | 0 | 7 |
|  | SA | 34 | 113 | 8 | 61 |
|  | A | 591 | 803 | 384 | 601 |
| Sex | F | 348 | 503 | 185 | 341 |
|  | M | 303 | 464 | 236 | 393 |
| Body Condition Score | 1 | 38 | 60 | 27 | 51 |
|  | 2 | 87 | 139 | 68 | 128 |
|  | 3 | 228 | 361 | 155 | 301 |
|  | 4 | 157 | 221 | 94 | 142 |
|  | 5 | 133 | 175 | 71 | 99 |
| Pregnant (Adult females) | Not Pregnant | 171 | 221 | 64 | 119 |
|  | Pregnant | 143 | 184 | 105 | 165 |
| Lactating (Adult females) | Not lactating | 201 | 267 | 131 | 217 |
|  | Lactating | 113 | 138 | 38 | 67 |
| PCR result | Negative | 617 | 929 | 421 | 734 |
|  | Positive | 34 | 38 | 0 | 0 |

**Supplementary Figure S1:** Frequency distribution histograms of HeV sG binding assay ln(MFI) values for each species.

**BFF GHFF LRFF**

BFF = black flying fox (n = 958); GHFF = grey-headed flying fox (n = 734); LRFF = little red flying fox (n = 205).

The red lines correspond to the predictive posterior means generated from the fitted mixture model, fitted to each species independently.

**Supplementary Figure S2:** Plots of the posterior mean (and 95% credible intervals) for the relative probability of a given HeV sG binding assay lnMFI value belonging to the seronegative, rather than seropositive groups for each species (generated from the fitted mixture model, fitted to all species independently).

**BFF GHFF LRFF**

The red lines indicate the posterior mean (and 95% credible intervals) for the cutoffs (the x-axis intercept) at which individuals with MFI values above this cutoff were >95% likely to be seropositive.

**Supplementary Figure S3:** The size and species composition of the flying-fox colony sampled at Boonah in southeast Queensland, June 2013 to June 2014.


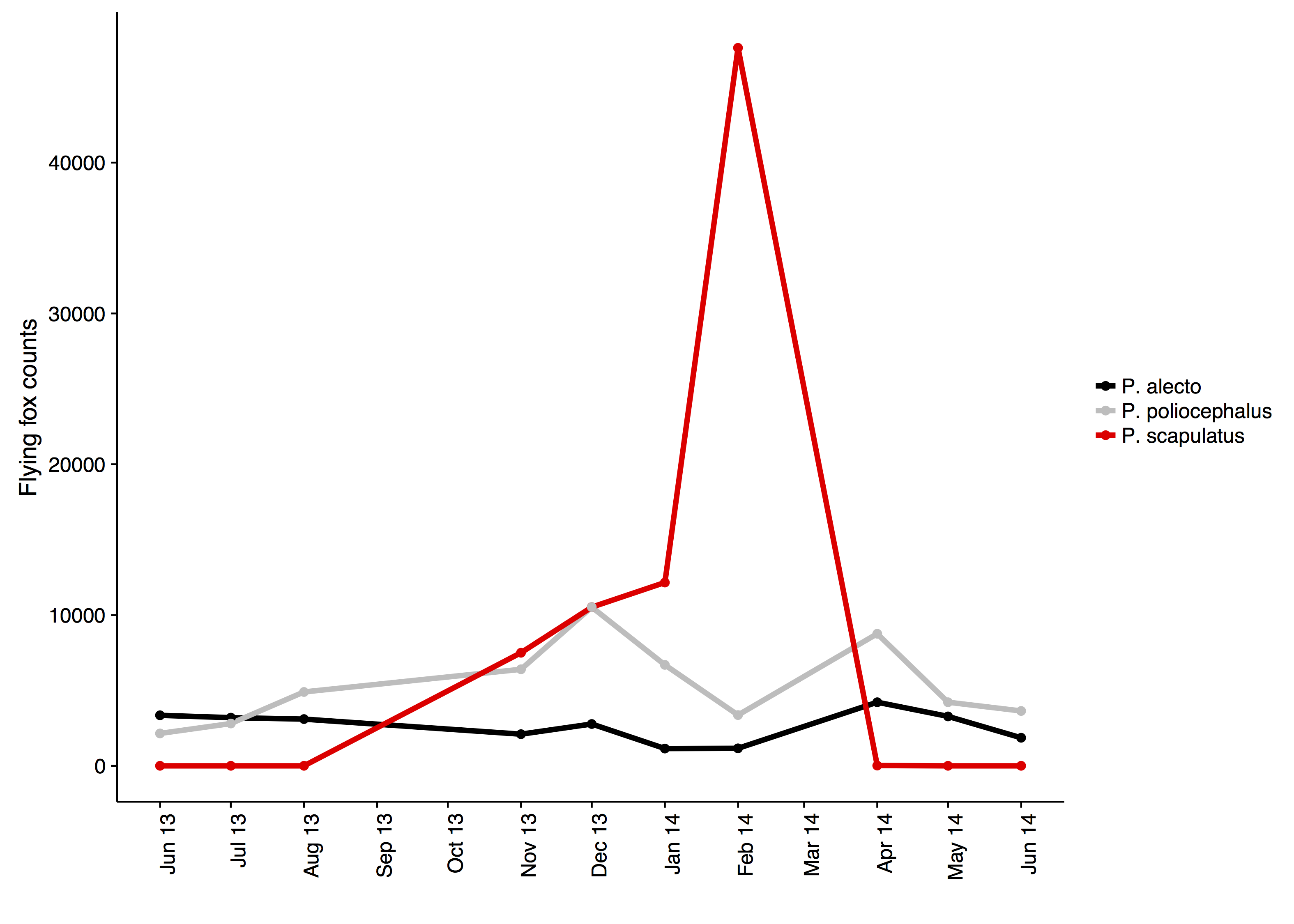


*P. alecto* = black flying-fox, *P. poliocephalus* = grey-headed flying-fox, *P. scapulatus* = Little red flying-fox.

**Supplementary Figure S4:** Hendra virus antibody titres (lnMFI) in adult *P. alecto* (black flying-fox) by sex and reproductive status.

**Supplementary Figure S5:** Hendra virus antibody titres (lnMFI) in adult *P. poliocephalus* (grey-headed flying-fox) by sex and reproductive status.

**Supplementary Figure S6:** Mean temporal lnMFI values across age cohorts in seropositive *P. alecto* (black flying-fox).


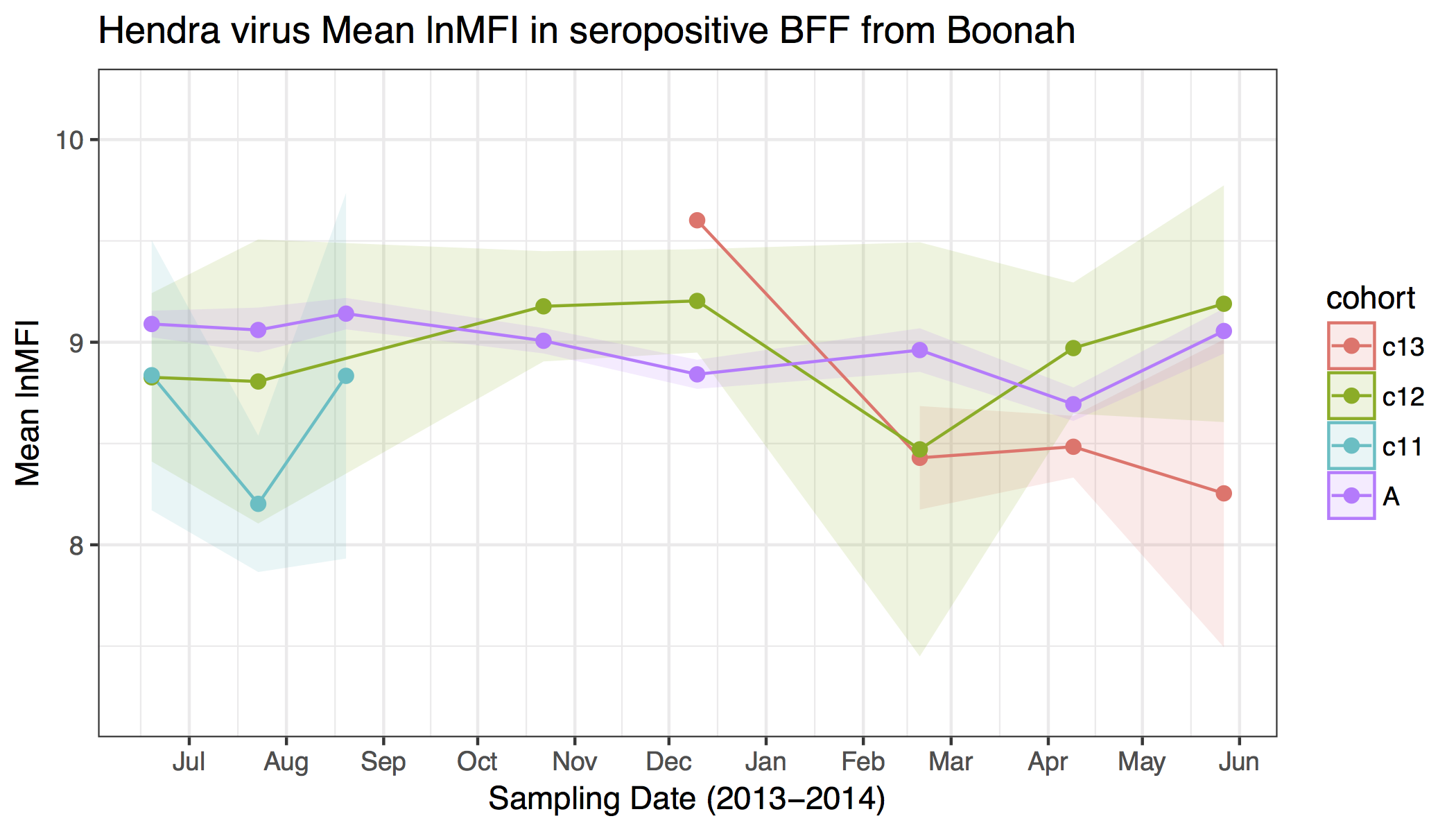


**Supplementary Figure S7:** Mean temporal lnMFI values across age cohorts in seropositive *P. poliocephalus* (grey-headed flying-fox).

**Supplementary Data S1:** List of GLMM model outputs

Terms:

pos_animal HeV RNA detection across any sample type (binary)

u_pcr HeV RNA detection in urine (binary)

s_pcr HeV RNA detection in serum (binary)

hev_pos95 HeV antibody detection in serum (binary)

ln_Serol_HeV_MFI HeV antibody titre in serum – natural log of the median fluorescence intensity (MFI) (continuous)

accession_mid Mid-point date of each sampling session

age Age class

cohort Age categorized by birth cohort

sex Sex

bcs Body condition score

pregnant Palpably pregnant

lactating Milk present in nipples

HeV RNA detection – across any sample type:

pos_animal ~ accession_mid + age + sex + accession_mid.age

mean deviance approx

Change d.f. deviance deviance ratio chi pr

+ accession_mid 7 34.6509 4.9501 4.95 <.001

+ age 2 8.7572 4.3786 4.38 0.013

+ sex 1 0.4915 0.4915 0.49 0.483

+ accession_mid.age 13 4.2400 0.3262 0.33 0.988

Residual 988 301.3819 0.3050

Total 1011 349.5215 0.3457

pos_animal ~ accession_mid + age + sex + accession_mid.age + bcs

mean deviance approx

Change d.f. deviance deviance ratio chi pr

+ accession_mid 7 34.4435 4.9205 4.92 <.001

+ age 2 8.8376 4.4188 4.42 0.012

+ sex 1 0.4395 0.4395 0.44 0.507

+ accession_mid.age 13 4.0596 0.3123 0.31 0.991

+ bcs 1 6.7200 6.7200 6.72 0.010

Residual 975 293.9977 0.3015

Total 999 348.4980 0.3488

pos_animal ~ accession_mid + age + sex + accession_mid.age + hev_pos95

mean deviance approx

Change d.f. deviance deviance ratio chi pr

+ accession_mid 7 33.9470 4.8496 4.85 <.001

+ age 2 8.2671 4.1336 4.13 0.016

+ sex 1 0.2120 0.2120 0.21 0.645

+ accession_mid.age 13 4.0308 0.3101 0.31 0.991

+ hev_pos95 1 14.0955 14.0955 14.10 <.001

Residual 942 259.9170 0.2759

Total 966 320.4693 0.3317

pos_animal ~ accession_mid + pregnant

mean deviance approx

Change d.f. deviance deviance ratio chi pr

+ accession_mid 7 18.6634 2.6662 2.67 0.009

+ pregnant 1 0.4809 0.4809 0.48 0.488

Residual 414 153.7697 0.3714

Total 422 172.9139 0.4097

pos_animal ~ accession_mid + lactating

mean deviance approx

Change d.f. deviance deviance ratio chi pr

+ accession_mid 7 18.6634 2.6662 2.67 0.009

+ lactating 1 0.5222 0.5222 0.52 0.470

Residual 414 153.7283 0.3713

Total 422 172.9139 0.4097

pos_animal ~ accession_mid + cohort + sex + accession_mid.cohort

mean deviance approx

Change d.f. deviance deviance ratio chi pr

+ accession_mid 7 34.6509 4.9501 4.95 <.001

+ cohort 3 7.2293 2.4098 2.41 0.065

+ sex 1 0.7105 0.7105 0.71 0.399

+ accession_mid.cohort 12 5.5488 0.4624 0.46 0.937

Residual 988 301.3819 0.3050

Total 1011 349.5215 0.3457

pos_animal ~ accession_mid + cohort + sex + accession_mid.cohort + bcs

mean deviance approx

Change d.f. deviance deviance ratio chi pr

+ accession_mid 7 34.4435 4.9205 4.92 <.001

+ cohort 3 7.3549 2.4516 2.45 0.061

+ sex 1 0.6494 0.6494 0.65 0.420

+ accession_mid.cohort 12 5.3325 0.4444 0.44 0.946

+ bcs 1 6.7200 6.7200 6.72 0.010

Residual 975 293.9977 0.3015

Total 999 348.4980 0.3488

pos_animal ~ accession_mid + cohort + sex + accession_mid.cohort + hev_pos95

mean deviance approx

Change d.f. deviance deviance ratio chi pr

+ accession_mid 7 33.9470 4.8496 4.85 <.001

+ cohort 3 6.5896 2.1965 2.20 0.086

+ sex 1 0.3945 0.3945 0.39 0.530

+ accession_mid.cohort 12 5.5257 0.4605 0.46 0.938

+ hev_pos95 1 14.0955 14.0955 14.10 <.001

Residual 942 259.9170 0.2759

Total 966 320.4693 0.3317

pos_animal ~ accession_mid + pregnant

mean deviance approx

Change d.f. deviance deviance ratio chi pr

+ accession_mid 7 18.6634 2.6662 2.67 0.009

+ pregnant 1 0.4809 0.4809 0.48 0.488

Residual 414 153.7697 0.3714

Total 422 172.9139 0.4097

pos_animal ~ accession_mid + lactating

mean deviance approx

Change d.f. deviance deviance ratio chi pr

+ accession_mid 7 18.6634 2.6662 2.67 0.009

+ lactating 1 0.5222 0.5222 0.52 0.470

Residual 414 153.7283 0.3713

Total 422 172.9139 0.4097

HeV RNA detection – in urine:

u_pcr ~ accession_mid + age + sex + accession_mid.age

mean deviance approx

Change d.f. deviance deviance ratio chi pr

+ accession_mid 7 22.6513 3.2359 3.24 0.002

+ age 2 3.2547 1.6274 1.63 0.196

+ sex 1 0.0433 0.0433 0.04 0.835

+ accession_mid.age 12 12.5807 1.0484 1.05 0.400

Residual 535 171.6849 0.3209

Total 557 210.2149 0.3774

u_pcr ~ accession_mid + age + sex + accession_mid.age + bcs

mean deviance approx

Change d.f. deviance deviance ratio chi pr

+ accession_mid 7 22.4957 3.2137 3.21 0.002

+ age 2 3.2737 1.6369 1.64 0.195

+ sex 1 0.0332 0.0332 0.03 0.855

+ accession_mid.age 12 12.4679 1.0390 1.04 0.409

+ bcs 1 1.6906 1.6906 1.69 0.194

Residual 529 169.7745 0.3209

Total 552 209.7356 0.3800

u_pcr ~ accession_mid + age + sex + accession_mid.age + hev_pos95

mean deviance approx

Change d.f. deviance deviance ratio chi pr

+ accession_mid 7 22.2606 3.1801 3.18 0.002

+ age 2 3.0959 1.5479 1.55 0.213

+ sex 1 0.1781 0.1781 0.18 0.673

+ accession_mid.age 12 12.0936 1.0078 1.01 0.438

+ hev_pos95 1 7.7418 7.7418 7.74 0.005

Residual 511 144.3783 0.2825

Total 534 189.7483 0.3553

u_pcr ~ accession_mid + pregnant

mean deviance approx

Change d.f. deviance deviance ratio chi pr

+ accession_mid 7 15.2431 2.1776 2.18 0.033

+ pregnant 1 2.2149 2.2149 2.21 0.137

Residual 210 81.1817 0.3866

Total 218 98.6398 0.4525

u_pcr ~ accession_mid + lactating

mean deviance approx

Change d.f. deviance deviance ratio chi pr

+ accession_mid 7 15.2431 2.1776 2.18 0.033

+ lactating 1 1.7970 1.7970 1.80 0.180

Residual 210 81.5997 0.3886

Total 218 98.6398 0.4525

u_pcr ~ accession_mid + cohort + sex + accession_mid.cohort

mean deviance approx

Change d.f. deviance deviance ratio chi pr

+ accession_mid 7 22.6513 3.2359 3.24 0.002

+ cohort 3 6.7117 2.2372 2.24 0.082

+ sex 1 0.1144 0.1144 0.11 0.735

+ accession_mid.cohort 11 9.0526 0.8230 0.82 0.617

Residual 535 171.6849 0.3209

Total 557 210.2149 0.3774

u_pcr ~ accession_mid + cohort + sex + accession_mid.cohort + bcs

mean deviance approx

Change d.f. deviance deviance ratio chi pr

+ accession_mid 7 22.4957 3.2137 3.21 0.002

+ cohort 3 6.6961 2.2320 2.23 0.082

+ sex 1 0.0996 0.0996 0.10 0.752

+ accession_mid.cohort 11 8.9791 0.8163 0.82 0.624

+ bcs 1 1.6906 1.6906 1.69 0.194

Residual 529 169.7745 0.3209

Total 552 209.7356 0.3800

u_pcr ~ accession_mid + cohort + sex + accession_mid.cohort + hev_pos95

mean deviance approx

Change d.f. deviance deviance ratio chi pr

+ accession_mid 7 22.2606 3.1801 3.18 0.002

+ cohort 3 7.2881 2.4294 2.43 0.063

+ sex 1 0.0307 0.0307 0.03 0.861

+ accession_mid.cohort 11 8.0487 0.7317 0.73 0.709

+ hev_pos95 1 7.7418 7.7418 7.74 0.005

Residual 511 144.3783 0.2825

Total 534 189.7483 0.3553

u_pcr ~ accession_mid + pregnant

mean deviance approx

Change d.f. deviance deviance ratio chi pr

+ accession_mid 7 15.2431 2.1776 2.18 0.033

+ pregnant 1 2.2149 2.2149 2.21 0.137

Residual 210 81.1817 0.3866

Total 218 98.6398 0.4525

u_pcr ~ accession_mid + lactating

mean deviance approx

Change d.f. deviance deviance ratio chi pr

+ accession_mid 7 15.2431 2.1776 2.18 0.033

+ lactating 1 1.7970 1.7970 1.80 0.180

Residual 210 81.5997 0.3886

Total 218 98.6398 0.4525

HeV RNA detection – in serum:

s_pcr ~ accession_mid + age + sex + accession_mid.age

mean deviance approx

Change d.f. deviance deviance ratio chi pr

+ accession_mid 7 19.88450 2.84064 2.84 0.006

+ age 2 6.90915 3.45457 3.45 0.032

+ sex 1 5.31014 5.31014 5.31 0.021

+ accession_mid.age 13 1.17214 0.09016 0.09 1.000

Residual 976 87.81955 0.08998

Total 999 121.09547 0.12122

s_pcr ~ accession_mid + age + sex + accession_mid.age + bcs

mean deviance approx

Change d.f. deviance deviance ratio chi pr

+ accession_mid 7 19.85655 2.83665 2.84 0.006

+ age 2 6.86590 3.43295 3.43 0.032

+ sex 1 5.27928 5.27928 5.28 0.022

+ accession_mid.age 13 1.17610 0.09047 0.09 1.000

+ bcs 1 3.16347 3.16347 3.16 0.075

Residual 964 84.50947 0.08767

Total 988 120.85078 0.12232

s_pcr ~ accession_mid + age + sex + accession_mid.age + hev_pos95

mean deviance approx

Change d.f. deviance deviance ratio chi pr

+ accession_mid 7 18.37622 2.62517 2.63 0.010

+ age 2 8.88646 4.44323 4.44 0.012

+ sex 1 3.84328 3.84328 3.84 0.050

+ accession_mid.age 13 1.31538 0.10118 0.10 1.000

+ hev_pos95 1 0.41375 0.41375 0.41 0.520

Residual 936 69.15386 0.07388

Total 960 101.98895 0.10624

s_pcr ~ accession_mid + pregnant

mean deviance approx

Change d.f. deviance deviance ratio chi pr

+ accession_mid 7 14.8023 2.1146 2.11 0.039

+ pregnant 1 0.2187 0.2187 0.22 0.640

Residual 409 56.1151 0.1372

Total 417 71.1361 0.1706

s_pcr ~ accession_mid + lactating

mean deviance approx

Change d.f. deviance deviance ratio chi pr

+ accession_mid 7 14.8023 2.1146 2.11 0.039

+ lactating 1 0.5743 0.5743 0.57 0.449

Residual 409 55.7595 0.1363

Total 417 71.1361 0.1706

s_pcr ~ accession_mid + cohort + sex + accession_mid.cohort

mean deviance approx

Change d.f. deviance deviance ratio chi pr

+ accession_mid 7 19.88450 2.84064 2.84 0.006

+ cohort 3 4.91549 1.63850 1.64 0.178

+ sex 1 5.63184 5.63184 5.63 0.018

+ accession_mid.cohort 12 2.84410 0.23701 0.24 0.997

Residual 976 87.81955 0.08998

Total 999 121.09547 0.12122

s_pcr ~ accession_mid + cohort + sex + accession_mid.cohort + bcs

mean deviance approx

Change d.f. deviance deviance ratio chi pr

+ accession_mid 7 19.85655 2.83665 2.84 0.006

+ cohort 3 4.86594 1.62198 1.62 0.182

+ sex 1 5.59842 5.59842 5.60 0.018

+ accession_mid.cohort 12 2.85692 0.23808 0.24 0.996

+ bcs 1 3.16347 3.16347 3.16 0.075

Residual 964 84.50947 0.08767

Total 988 120.85078 0.12232

s_pcr ~ accession_mid + cohort + sex + accession_mid.cohort + hev_pos95

mean deviance approx

Change d.f. deviance deviance ratio chi pr

+ accession_mid 7 18.37622 2.62517 2.63 0.010

+ cohort 3 6.63014 2.21005 2.21 0.085

+ sex 1 4.16375 4.16375 4.16 0.041

+ accession_mid.cohort 12 3.25123 0.27094 0.27 0.993

+ hev_pos95 1 0.41375 0.41375 0.41 0.520

Residual 936 69.15386 0.07388

Total 960 101.98895 0.10624

s_pcr ~ accession_mid + pregnant

mean deviance approx

Change d.f. deviance deviance ratio chi pr

+ accession_mid 7 14.8023 2.1146 2.11 0.039

+ pregnant 1 0.2187 0.2187 0.22 0.640

Residual 409 56.1151 0.1372

Total 417 71.1361 0.1706

s_pcr ~ accession_mid + lactating

mean deviance approx

Change d.f. deviance deviance ratio chi pr

+ accession_mid 7 14.8023 2.1146 2.11 0.039

+ lactating 1 0.5743 0.5743 0.57 0.449

Residual 409 55.7595 0.1363

Total 417 71.1361 0.1706

HeV antibody detection:

hev_pos95 ~ accession_mid + age + species + sex + accession_mid.age

mean deviance approx

Change d.f. deviance deviance ratio chi pr

+ accession_mid 7 31.016 4.431 4.43 <.001

+ age 2 131.808 65.904 65.90 <.001

+ species 1 22.969 22.969 22.97 <.001

+ sex 1 0.149 0.149 0.15 0.700

+ accession_mid.age 13 35.300 2.715 2.72 <.001

Residual 1676 2020.131 1.205

Total 1700 2241.373 1.318

hev_pos95 ~ accession_mid + age + species + sex + accession_mid.age + bcs

mean deviance approx

Change d.f. deviance deviance ratio chi pr

+ accession_mid 7 30.193 4.313 4.31 <.001

+ age 2 124.544 62.272 62.27 <.001

+ species 1 22.182 22.182 22.18 <.001

+ sex 1 0.179 0.179 0.18 0.672

+ accession_mid.age 13 35.370 2.721 2.72 <.001

+ bcs 1 10.910 10.910 10.91 <.001

Residual 1651 1985.166 1.202

Total 1676 2208.546 1.318

hev_pos95 ~ accession_mid + species + accession_mid.species + pregnant

mean deviance approx

Change d.f. deviance deviance ratio chi pr

+ accession_mid 7 10.619 1.517 1.52 0.156

+ species 1 22.348 22.348 22.35 <.001

+ accession_mid.species 7 8.254 1.179 1.18 0.311

+ pregnant 1 1.204 1.204 1.20 0.273

Residual 672 798.156 1.188

Total 688 840.581 1.222

hev_pos95 ~ accession_mid + species + accession_mid.species + lactating

mean deviance approx

Change d.f. deviance deviance ratio chi pr

+ accession_mid 7 10.619 1.517 1.52 0.156

+ species 1 22.348 22.348 22.35 <.001

+ accession_mid.species 7 8.254 1.179 1.18 0.311

+ lactating 1 1.259 1.259 1.26 0.262

Residual 672 798.100 1.188

Total 688 840.581 1.222

hev_pos95 ~ accession_mid + cohort + species + sex + accession_mid.cohort

mean deviance approx

Change d.f. deviance deviance ratio chi pr

+ accession_mid 7 31.016 4.431 4.43 <.001

+ cohort 3 141.953 47.318 47.32 <.001

+ species 1 25.192 25.192 25.19 <.001

+ sex 1 0.262 0.262 0.26 0.608

+ accession_mid.cohort 12 22.819 1.902 1.90 0.029

Residual 1676 2020.131 1.205

Total 1700 2241.373 1.318

hev_pos95 ~ accession_mid + cohort + species + sex + accession_mid.cohort + bcs

mean deviance approx

Change d.f. deviance deviance ratio chi pr

+ accession_mid 7 30.193 4.313 4.31 <.001

+ cohort 3 135.168 45.056 45.06 <.001

+ species 1 24.383 24.383 24.38 <.001

+ sex 1 0.297 0.297 0.30 0.586

+ accession_mid.cohort 12 22.427 1.869 1.87 0.033

+ bcs 1 10.910 10.910 10.91 <.001

Residual 1651 1985.166 1.202

Total 1676 2208.546 1.318

hev_pos95 ~ accession_mid + species + accession_mid.species + pregnant

mean deviance approx

Change d.f. deviance deviance ratio chi pr

+ accession_mid 7 10.619 1.517 1.52 0.156

+ species 1 22.348 22.348 22.35 <.001

+ accession_mid.species 7 8.254 1.179 1.18 0.311

+ cohort 0 0.000 *

+ pregnant 1 1.204 1.204 1.20 0.273

Residual 672 798.156 1.188

Total 688 840.581 1.222

hev_pos95 ~ accession_mid + species + accession_mid.species + lactating

mean deviance approx

Change d.f. deviance deviance ratio chi pr

+ accession_mid 7 10.619 1.517 1.52 0.156

+ species 1 22.348 22.348 22.35 <.001

+ accession_mid.species 7 8.254 1.179 1.18 0.311

+ cohort 0 0.000 *

+ lactating 1 1.259 1.259 1.26 0.262

Residual 672 798.100 1.188

Total 688 840.581 1.222

HeV antibody titre:

ln_Serol_HeV_MFI ~ accession_mid + age + species + sex + accession_mid.age

Change d.f. s.s. m.s. v.r. F pr.

+ accession_mid 7 197.208 28.173 7.99 <.001

+ age 2 637.286 318.643 90.37 <.001

+ species 1 266.297 266.297 75.53 <.001

+ sex 1 6.720 6.720 1.91 0.168

+ accession_mid.age 13 88.449 6.804 1.93 0.023

Residual 1652 5824.794 3.526

Total 1676 7020.754 4.189

ln_Serol_HeV_MFI ~ accession_mid + age + species + sex + accession_mid.age + bcs

Change d.f. s.s. m.s. v.r. F pr.

+ accession_mid 7 197.208 28.173 8.05 <.001

+ age 2 637.286 318.643 90.99 <.001

+ species 1 266.297 266.297 76.04 <.001

+ sex 1 6.720 6.720 1.92 0.166

+ accession_mid.age 13 88.449 6.804 1.94 0.022

+ bcs 1 43.241 43.241 12.35 <.001

Residual 1651 5781.554 3.502

Total 1676 7020.754 4.189

ln_Serol_HeV_MFI ~ accession_mid + age + species + sex + accession_mid.age + pos_animal

Change d.f. s.s. m.s. v.r. F pr.

+ accession_mid 7 197.208 28.173 8.07 <.001

+ age 2 637.286 318.643 91.30 <.001

+ species 1 266.297 266.297 76.30 <.001

+ sex 1 6.720 6.720 1.93 0.165

+ accession_mid.age 13 88.449 6.804 1.95 0.022

+ pos_animal 1 62.603 62.603 17.94 <.001

Residual 1651 5762.191 3.490

Total 1676 7020.754 4.189

ln_Serol_HeV_MFI ~ accession_mid + species + pregnant

Change d.f. s.s. m.s. v.r. F pr.

+ accession_mid 7 89.899 12.843 3.25 0.002

+ species 1 163.739 163.739 41.42 <.001

+ pregnant 1 7.863 7.863 1.99 0.159

Residual 679 2684.122 3.953

Total 688 2945.624 4.281

ln_Serol_HeV_MFI ~ accession_mid + species + lactating

Change d.f. s.s. m.s. v.r. F pr.

+ accession_mid 7 89.899 12.843 3.24 0.002

+ species 1 163.739 163.739 41.37 <.001

+ lactating 1 4.575 4.575 1.16 0.283

Residual 679 2687.410 3.958

Total 688 2945.624 4.281

ln_Serol_HeV_MFI ~ accession_mid + cohort + species + sex + accession_mid.cohort

Change d.f. s.s. m.s. v.r. F pr.

+ accession_mid 7 197.208 28.173 7.99 <.001

+ cohort 3 655.250 218.417 61.95 <.001

+ species 1 276.575 276.575 78.44 <.001

+ sex 1 7.377 7.377 2.09 0.148

+ accession_mid.cohort 12 59.550 4.962 1.41 0.155

Residual 1652 5824.794 3.526

Total 1676 7020.754 4.189

ln_Serol_HeV_MFI ~ accession_mid + cohort + species + sex + accession_mid.cohort + bcs

Change d.f. s.s. m.s. v.r. F pr.

+ accession_mid 7 197.208 28.173 8.05 <.001

+ cohort 3 655.250 218.417 62.37 <.001

+ species 1 276.575 276.575 78.98 <.001

+ sex 1 7.377 7.377 2.11 0.147

+ accession_mid.cohort 12 59.550 4.962 1.42 0.151

+ bcs 1 43.241 43.241 12.35 <.001

Residual 1651 5781.554 3.502

Total 1676 7020.754 4.189

ln_Serol_HeV_MFI ~ accession_mid + cohort + species + sex + accession_mid.cohort + pos_animal

Change d.f. s.s. m.s. v.r. F pr.

+ accession_mid 7 197.208 28.173 8.07 <.001

+ cohort 3 655.250 218.417 62.58 <.001

+ species 1 276.575 276.575 79.25 <.001

+ sex 1 7.377 7.377 2.11 0.146

+ accession_mid.cohort 12 59.550 4.962 1.42 0.149

+ pos_animal 1 62.603 62.603 17.94 <.001

Residual 1651 5762.191 3.490

Total 1676 7020.754 4.189

ln_Serol_HeV_MFI ~ accession_mid + species + pregnant

Change d.f. s.s. m.s. v.r. F pr.

+ accession_mid 7 89.899 12.843 3.25 0.002

+ species 1 163.739 163.739 41.42 <.001

+ pregnant 1 7.863 7.863 1.99 0.159

Residual 679 2684.122 3.953

Total 688 2945.624 4.281

ln_Serol_HeV_MFI ~ accession_mid + species + lactating

Change d.f. s.s. m.s. v.r. F pr.

+ accession_mid 7 89.899 12.843 3.24 0.002

+ species 1 163.739 163.739 41.37 <.001

+ lactating 1 4.575 4.575 1.16 0.283

Residual 679 2687.410 3.958

Total 688 2945.624 4.281

HeV antibody titre - seropositive animals only:

ln_Serol_HeV_MFI ~ accession_mid + age + species + sex + accession_mid.age

Change d.f. s.s. m.s. v.r. F pr.

+ accession_mid 7 16.7013 2.3859 4.76 <.001

+ age 2 6.8401 3.4200 6.83 0.001

+ species 1 22.8762 22.8762 45.66 <.001

+ sex 1 31.2375 31.2375 62.35 <.001

+ accession_mid.age 12 6.4343 0.5362 1.07 0.382

Residual 1034 518.0514 0.5010

Total 1057 602.1408 0.5697

ln_Serol_HeV_MFI ~ accession_mid + age + species + sex + accession_mid.age + bcs

Change d.f. s.s. m.s. v.r. F pr.

+ accession_mid 7 16.7013 2.3859 4.76 <.001

+ age 2 6.8401 3.4200 6.82 0.001

+ species 1 22.8762 22.8762 45.62 <.001

+ sex 1 31.2375 31.2375 62.29 <.001

+ accession_mid.age 12 6.4343 0.5362 1.07 0.383

+ bcs 1 0.0375 0.0375 0.07 0.785

Residual 1033 518.0140 0.5015

Total 1057 602.1408 0.5697

ln_Serol_HeV_MFI ~ accession_mid + age + species + sex + accession_mid.age + pos_animal

Change d.f. s.s. m.s. v.r. F pr.

+ accession_mid 7 16.7013 2.3859 4.86 <.001

+ age 2 6.8401 3.4200 6.96 <.001

+ species 1 22.8762 22.8762 46.58 <.001

+ sex 1 31.2375 31.2375 63.61 <.001

+ accession_mid.age 12 6.4343 0.5362 1.09 0.363

+ pos_animal 1 10.7420 10.7420 21.87 <.001

Residual 1033 507.3094 0.4911

Total 1057 602.1408 0.5697

ln_Serol_HeV_MFI ~ accession_mid + species + pregnant

Change d.f. s.s. m.s. v.r. F pr.

+ accession_mid 7 20.3236 2.9034 6.63 <.001

+ species 1 5.9185 5.9185 13.52 <.001

+ pregnant 1 0.1500 0.1500 0.34 0.559

Residual 473 207.1350 0.4379

Total 482 233.5271 0.4845

ln_Serol_HeV_MFI ~ accession_mid + species + lactating

Change d.f. s.s. m.s. v.r. F pr.

+ accession_mid 7 20.3236 2.9034 6.65 <.001

+ species 1 5.9185 5.9185 13.56 <.001

+ lactating 1 0.8074 0.8074 1.85 0.174

Residual 473 206.4776 0.4365

Total 482 233.5271 0.4845

ln_Serol_HeV_MFI ~ accession_mid + cohort + species + sex + accession_mid.cohort

Change d.f. s.s. m.s. v.r. F pr.

+ accession_mid 7 16.7013 2.3859 4.76 <.001

+ cohort 3 9.9810 3.3270 6.64 <.001

+ species 1 22.7938 22.7938 45.50 <.001

+ sex 1 29.9697 29.9697 59.82 <.001

+ accession_mid.cohort 11 4.6436 0.4221 0.84 0.597

Residual 1034 518.0514 0.5010

Total 1057 602.1408 0.5697

ln_Serol_HeV_MFI ~ accession_mid + cohort + species + sex + accession_mid.cohort + bcs

Change d.f. s.s. m.s. v.r. F pr.

+ accession_mid 7 16.7013 2.3859 4.76 <.001

+ cohort 3 9.9810 3.3270 6.63 <.001

+ species 1 22.7938 22.7938 45.45 <.001

+ sex 1 29.9697 29.9697 59.76 <.001

+ accession_mid.cohort 11 4.6436 0.4221 0.84 0.598

+ bcs 1 0.0375 0.0375 0.07 0.785

Residual 1033 518.0140 0.5015

Total 1057 602.1408 0.5697

ln_Serol_HeV_MFI ~ accession_mid + cohort + species + sex + accession_mid.cohort + pos_animal

Change d.f. s.s. m.s. v.r. F pr.

+ accession_mid 7 16.7013 2.3859 4.86 <.001

+ cohort 3 9.9810 3.3270 6.77 <.001

+ species 1 22.7938 22.7938 46.41 <.001

+ sex 1 29.9697 29.9697 61.03 <.001

+ accession_mid.cohort 11 4.6436 0.4221 0.86 0.580

+ pos_animal 1 10.7420 10.7420 21.87 <.001

Residual 1033 507.3094 0.4911

Total 1057 602.1408 0.5697

ln_Serol_HeV_MFI ~ accession_mid + species + pregnant

Change d.f. s.s. m.s. v.r. F pr.

+ accession_mid 7 20.3236 2.9034 6.63 <.001

+ species 1 5.9185 5.9185 13.52 <.001

+ pregnant 1 0.1500 0.1500 0.34 0.559

Residual 473 207.1350 0.4379

Total 482 233.5271 0.4845

ln_Serol_HeV_MFI ~ accession_mid + species + lactating

Change d.f. s.s. m.s. v.r. F pr.

+ accession_mid 7 20.3236 2.9034 6.65 <.001

+ species 1 5.9185 5.9185 13.56 <.001

+ lactating 1 0.8074 0.8074 1.85 0.174

Residual 473 206.4776 0.4365

Total 482 233.5271 0.4845
